# Effects of Combination Therapy with Intravitreal Ranibizumab and Tissue Plasminogen Activator for Neovascular Age-Related Macular Degeneration

**DOI:** 10.3390/jcm13082417

**Published:** 2024-04-21

**Authors:** Michiko Ando, Aki Kato, Masayo Kimura, Shuntaro Ogura, Soichiro Kuwayama, Aoi Kominami, Satoshi Kuwayama, Tomohiro Obayashi, Ryota Ando, Takafumi Monoe, Hiroshi Morita, Tsutomu Yasukawa

**Affiliations:** 1 Department of Ophthalmology and Visual Science, Nagoya City University Graduate School of Medical Sciences, 1 Kawasumi, Mizuho-cho, Mizuho-ku, Nagoya 467-8601, Japan; ando831388@gmail.com (M.A.); love4u030301@gmail.com (M.K.); s-ogura@med.nagoya-cu.ac.jp (S.O.); gifu8847@yahoo.co.jp (S.K.); pink299pu2pu2@gmail.com (A.K.); manga.anime.takkyuu@gmail.com (S.K.); t_o_gemgem@yahoo.co.jp (T.O.); steve-0515@outlook.com (R.A.); whitedwarf3000@gmail.com (T.M.); hiroshi1966morita@au.com (H.M.); yasukawa@med.nagoya-cu.ac.jp (T.Y.); 2 Department of Ophthalmology, Ogaki Tokushukai Hospital, 6-85-1 Hayashimachi, Ogaki 503-0015, Japan; 3 Department of Ophthalmology, Inazawa Kosei Hospital, 7 Sobuechojitchono, Inazawa 495-8531, Japan; 4 Department of Ophthalmology, Daido Hospital, 9 Hakusui-cho, Minami-ku, Nagoya 457-8511, Japan; 5 Department of Ophthalmology, Nagoya City University East Medical Center, 1-2-23 Wakamizu, Chikusa-ku, Nagoya 464-8547, Japan; 6 Department of Ophthalmology, Central Japan International Medical Center, 1-1 Kenkonomachi, Minokamo 505-8510, Japan

**Keywords:** neovascular age-related macular degeneration, anti-vascular endothelial growth factor therapy, subretinal hyper-reflective material, tissue plasminogen activator

## Abstract

**Background:** Subretinal hyper-reflective material (SHRM) sometimes causes vision loss in spite of anti-vascular endothelial growth factor (VEGF) therapy in eyes with neovascular age-related macular degeneration (nvAMD). We evaluated the impacts of combination therapy with intravitreal ranibizumab (IVR) and tissue plasminogen activator (tPA) in eyes with nvAMD accompanying SHRM. **Methods:** In total, 25 eyes of 25 patients (16 men and 9 women, 76.7 years old), who underwent IVR/tPA for nvAMD with SHRM and were followed up for at least 12 months, were retrospectively reviewed. In total, 15 eyes were treatment-naïve and 10 eyes had previous treatment for nvAMD. **Results:** In total, 16 eyes had type 2 macular neovascularization (MNV), 5 eyes type 1 MNV with fibrovascular pigment epithelial detachment and 4 eyes polypoidal choroidal vasculopathy. At month 12, SHRM regressed or reduced in 18 eyes (72%) and the best-corrected visual acuity (BCVA) improved in 6 eyes (24%) and was unchanged in 14 eyes (56%), while the mean BCVA was just stabilized. The mean central retinal thickness, macular volume and SHRM thickness significantly improved from 408 µm to 287 µm, from 11.9 mm^3^ to 9.6 mm^3^, from 369 µm to 165 µm, respectively (*p* < 0.01). **Conclusions:** The combination therapy with IVR/tPA for nvAMD with SHRM may help preserve vision by prompt regression of SHRM.

## 1. Introduction

Neovascular age-related macular degeneration (nvAMD) is a major cause of visual loss in elderly populations in developed countries [1], and the incidence in Japan has been increasing in recent years [2,3]. Since the efficacy and safety of intravitreal ranibizumab (IVR) (Lucentis^®^, Genentech Inc., South San Francisco, CA, USA) for nvAMD has been demonstrated [4,5], anti-vascular endothelial growth factor (VEGF) therapy is the first-line treatment for nvAMD. Subsequent approvals of aflibercept (Eylea^®^, Regeneron Pharmaceutical Inc., Tarrytown, NY, USA) [6], brolucizumab [7] (Beovu^®^ Novartis, East Hanover, NJ, USA) and faricimab [8] (Vabysmo^®^ Roche, Basel, Switzerland) expanded the options for anti-VEGF therapy.

In initial clinical trials [4,5,6], the effectiveness of a fixed monthly dosing regimen was assessed. the requirement for monthly injections posed significant financial and mental burdens for the patients. Subsequent research aimed to develop dosing schedules that reduce the injection frequency while maintaining good visual outcomes. One such regimen is the pro re nata (PRN) approach [9], in which treatment is administered based on signs of disease activity. In contrast, the treat-and-extend (TAE) regimen [10] involves adjusting the treatment intervals based on disease activity without the need for monthly assessments. Moreover, the TAE regimen seeks to proactively manage patients by identifying individual relapse patterns. Studies have shown that the TAE regimen is comparable to the fixed regimen and superior to the PRN regimen [11]. Consequently, it is commonly used alongside PRN [12]. Recently approved brolucizumab (Beovu, Novartis, Basel, Switzerland) [7] and faricimab (Vabysmo, Genentech Inc.) [8] have shown efficacy with intervals of 12 to 16 weeks, which represents significant progress in extending the intervals and reducing the treatment burden on patients.

The use of gene therapy via subretinal injection of recombinant adeno-associated vectors [13] or intravitreous injection of AAV2-sFLT01 [14], and transplantation of retinal pigment epithelial (RPE) cells derived from induced pluripotent stem cells (iPSCs) [15] or HLA-matched allogeneic iPSC-derived retinal cells [16], have received considerable attention as potential treatments for nvAMD. However, the practical application of these therapies has been hindered by concerns regarding safety, versatility, and cost. Recently, port delivery systems for anti-VEGF drugs, ref. [17] have been explored and provide less burdensome treatment options. Nevertheless, the primary objective of these approaches is to enhance the sustained efficacy of anti-VEGF drugs over prolonged periods.

However, long-term outcomes from large trials have revealed the limitations of anti-VEGF treatment [18]. While anti-VEGF therapy effectively suppresses choroidal neovascularization (CNV) and inhibits hyperpermeability and resulting exudative changes, it does not cause regression of active fibrovascular neovascularization, such as fibrin-involved neovascularization, that has already developed. Consequently, the formation of fibrovascular scars in the macula can lead to severe visual impairment.

Subretinal hyperreflective material (SHRM) is identified as a homogeneous high-intensity signal located between the neurosensory retina and retinal pigment epithelium (RPE) on optical coherence tomography (OCT). SHRM reflects subretinal fibrinous and fibrovascular tissue complexes of type 2 macular neovascularization (MNV) as well as fibrin mass and subretinal hemorrhage observed in any type of MNV. SHRM is an important morphological biomarker, associated with fibrotic and non-fibrotic scar formation and is considered a risk factor for vision loss in spite of anti-VEGF therapy in eyes with nvAMD [19,20,21,22]. Jaffe et al. [18] evaluated the associations between morphologic features and 5-year visual acuity (VA) in the Comparison of Age-related Macular Degeneration Treatment Trials (CATT) and reported that the SHRM, thinner retina, greater CNV lesion area, and foveal center pathology and intraretinal fluid (IRF) were independently associated with worse VA. Kumar et al. [23] evaluated the morphology of the SHRM (reflectivity, shape, anterior, and posterior boundaries) and measured the SHRM height, width, and area at the fovea in eyes with nvAMD and reported that SHRM including a layered appearance, increased reflectivity, larger size, and hyperreflective spots was correlated with worse VA at the 12- and 24-week follow-up examinations. The baseline SHRM characteristics can help practitioners predict visual and morphologic prognosis and guide therapy.

Tissue plasminogen activator (tPA) is a serine protease with a catalytic ability to convert plasminogen to plasmin, a major fibrinolytic enzyme. tPA is clinically used for embolic or thrombotic stroke. tPA is also used as an adjuvant to displace submacular hemorrhages [24,25,26,27,28,29] in nvAMD or ruptured retinal macroaneurysm. In our previous investigation [24], 59% of patients who received tPA and sulfur hexafluoride (SF6) gas injection for submacular hemorrhages (SMH) secondary to AMD did not require supplementary interventions throughout the follow-up period. Additionally, in certain cases, notable regression of subretinal fibrinous masses occurred promptly after treatment and was potentially linked to the administration of tPA. Based on these findings, we deliberated on the use of intravitreal tPA in conjunction with ranibizumab for type 2 MNV. In a previous study [30], in eyes with type 2 MNV, rapid and pronounced regression or contraction of subretinal fibrinous or fibrovascular tissue complexes, coupled with their separation from the outer retina, was observed in eyes treated with the combined therapy of intravitreal ranibizumab and tPA (IVR/tPA) but not in those treated with IVR monotherapy. Additionally, the mean best-corrected VA (BCVA) in the IVR/tPA combination therapy group surpassed that of the anti-VEGF monotherapy group by month 6. These findings suggested that tPA may have the specific ability to cause regression of already formed subretinal fibrinous and fibrovascular tissue complexes in eyes with type 2 MNV while preserving the VA. Hence, we hypothesized that tPA/IVR may be useful for managing SHRM characterized predominantly by subretinal fibrinous and fibrovascular tissue complexes, fibrin masses, and subretinal hemorrhage.

The purpose of this study was to evaluate the impacts of the combination therapy with IVR/tPA in eyes with nvAMD accompanying SHRM.

## 2. Materials and Methods

### 2.1. Study Design and Ethics

This was a retrospective single-center observational study conducted in Nagoya City University Hospital. The institutional review board (IRB) of Nagoya City University Graduate School of Medical Science approved the study protocol (IRB approved number, 60-19-0222) which was conducted in compliance with the ethical guidelines of the Declaration of Helsinki and registered in UMIN-CTR (UMIN registration number, UMIN000046415). The requirement for informed consent was waived because of the retrospective observational nature of the study. The IRB of Nagoya City University Graduate School of Medical Science had approved the tPA/IVR treatment for nvAMD (IRB approved number, 41-12-0005). All patients provided informed, written consent. Patients were examined regularly for clinical findings and adverse events, which were reported annually to the IRB.

### 2.2. Patients

The study enrolled 25 eyes of 25 patients (16 men, 9 women) diagnosed with nvAMD accompanied by SHRM who underwent tPA/IVR at Nagoya City University Hospital between April 2009 and March 2021, who could be followed for at least 1 year, and necessary data were available. Cases of PCV with both SRH and hard exudates were excluded from this treatment due to the risk of exacerbating exudative changes by fibrin dissolution with tPA. Additionally, patients with large SRHs involving the fovea were not candidates for this treatment, because pneumatic displacement is more appropriate. Eyes with high myopia or a history of other vitreoretinal diseases such as retinal detachment, diabetic retinopathy, retinal vein occlusion, and uveitis also were excluded. Further, patients with less potential for VA improvement or those at risk of worsening systemic diseases also were excluded from receiving this treatment due to its unapproved status.

Treatment with IVR/tPA included topical instillation of 4% xylocaine under sterile conditions followed by intravitreal injection of tPA (40 kIU/100 µL of monteplase, Cleactor^®^, Eisai Co., Ltd., Tokyo, Japan) with a 30-gauge needle. After paracentesis to reduce intraocular pressure, intravitreal injection of ranibizumab (0.5 mg/50 µL) was administered. If intraocular pressure was elevated after IVR, additional paracenteses were performed.

The dosage of tPA was based on 40 kIU, as reported previously [31,32,33].

After IVR/tPA, patients were treated in the manner of a pro re nata (PRN) regimen with any anti-VEGF drugs.

### 2.3. Research and Analysis

The medical data assessed included the patient’s ages at the start of treatment of nvAMD, AMD subtypes, previous treatment history, additional treatments (anti-VEGF injections: IVR or aflibercept or any other treatment) and medical history. The ophthalmologic examination including the BCVA in decimal units and the central retinal thickness (CRT), macular volume (MV), and SHRM thickness measured by OCT (Cirrus OCT, Carl Zeiss, Dublin, CA, USA) evaluated at the first visit, just before IVR/tPA, 1, 3, 6 and 12 months after IVR/tPA. The CRT and MV were obtained from automatic measurements on OCT. The SHRM thickness, observed as a high-intensity signal between the neurosensory retina and RPE on OCT, was determined as the maximal diameter of the SHRM measured manually on OCT images. For cases in which OCT was performed within 1 week, the SHRM status was compared between the OCT images obtained immediately before and immediately after treatment.

### 2.4. Statistical Analysis

The analysis of the VA was performed by converting the values from decimal units to a logarithm of the minimum angle of resolution (logMAR) units. The changes of 30% or more SHRM thicknesses were defined as reduced and worsened. ‘Complete regression’ was defined as no SHRM observed on OCT. Changes of 0.3 or more in the BCVA were defined as improved or worsened. The differences in the BCVA, CRT, MV, and SHRM thickness before treatment and at each time point were compared using the Steel–Dwass test. The differences in the BCVA at month 12 among the SHRM statuses were compared using the Steel–Dwass test, the differences among the AMD subtypes were compared using Bonferroni’s test, and the differences between eyes with/without previous treatment were compared using the unpaired test. *p* < 0.05 was considered significant. Microsoft Excel Ver. 7 software (Microsoft Corporation, Redmond, WA, USA) was used for the statistical analyses.

## 3. Results

### 3.1. Patient Characteristics

The patient characteristics are shown in Table 1. In total, 25 eyes of 25 cases (16 men and 9 women; mean age, 76.7 ± 9.8 years) were evaluated. Regarding the medical history, 11 patients had no remarkable medical history, 8 had hypertension, 3 had cardiovascular disease, 4 had cerebrovascular disease, and 4 had diabetes (including duplicate cases). The AMD subtypes included type 2 macular neovascularization (MNV) in 16 eyes, type 1 MNV with fibrovascular pigment epithelial detachment (PED) in 5 eyes, and polypoidal choroidal vasculopathy (PCV) in 4 eyes.

Twenty-four eyes received 40 kIU, and one patient with a history of acute myocardial infarction received 20 kIU at the discretion of the attending physician. While 15 eyes were treatment naïve, the other 10 eyes were treated with anti-VEGF therapy, photodynamic therapy (PDT), or both. Of these 10 eyes, 4 eyes received IVR monotherapy, 3 eyes intravitreal aflibercept (IVA) monotherapy, 1 eye IVR and IVA, 1 eye IVR and PDT, and 1 eye IVR, IVA, and PDT. In the previous treatment group, six months before the start of treatment, the mean total number of anti-VEGF injections was 2.1.

### 3.2. Status of SHRM Immediately after Treatmen

**Figure 1:**
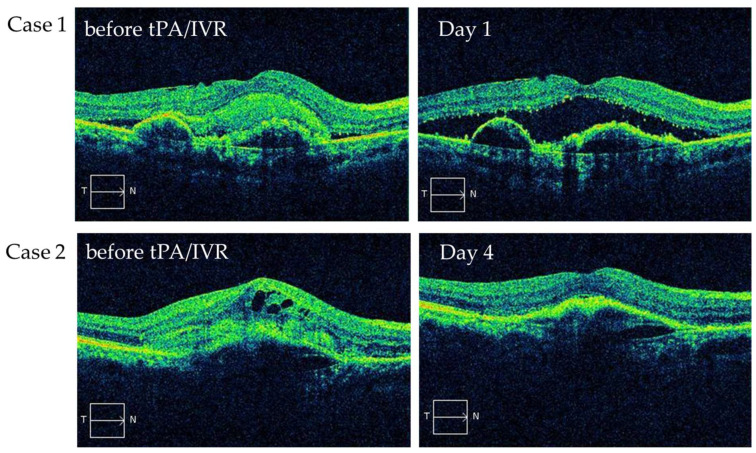
Changes in SHRM immediately after treatment. Fibrin disappeared on the next day (**case 1**), and exudative changes improved on day 4 (**case 2**).

In total, 8 of the 25 patients underwent OCT imaging the next day, and 1 underwent OCT imaging on day 4. The fibrin disappeared in all patients (Figure 1 Case1), and the size of the type 2 MNV decreased in affected patients. On day 4, the exudative changes improved (Figure 1 Case2).

### 3.3. Changes in Each Parameter

**Figure 2:**
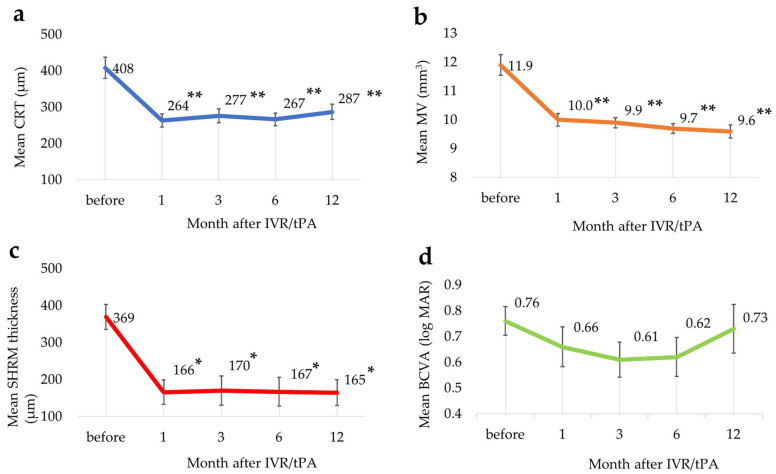
Changes in each parameter. The mean changes in central retinal thickness (CRT) (**a**), macular volume (MV) (**b**), subretinal hyperreflective material (SHRM) thickness (**c**) and the best-corrected visual acuity (BCVA) (**d**). * *p* < 0.05, ** *p* < 0.01, (Steel–Dwass test). bar = ±standard error of the mean.

The changes in each parameter are shown in Figure 2.

The mean CRTs at baseline and months 1, 3, 6, and 12 were 408 ± 148 μm, 264 ± 89 μm, 277 ± 95 μm, 267 ± 88 μm, and 287 ± 104 μm, respectively, the mean CRTs improved significantly at all time points after IVR/tPA compared with baseline (*p*-value < 0.01, Dunnett’s test) (Figure 2a). The mean MVs at baseline and months 1, 3, 6, and 12 were 11.9 ± 1.6 mm^3^, 10.0 ± 1.0 mm^3^, 9.9 ± 0.8 mm^3^, 9.7 ± 0.8 mm^3^, and 9.6 ± 1.0 mm^3^, respectively. The mean MVs also improved significantly (*p* < 0.01) at all time points after IVR/tPA compared with baseline (Figure 2b). The mean SHRM thicknesses at baseline and months 1, 3, 6, and 12 were 369 ± 163 μm, 166 ± 160 μm, 170 ± 190 μm, 167 ± 186 μm, and 165 ± 164 μm, respectively. The mean SHRM thickness also improved significantly (*p* < 0.05) at all time points after IVR/tPA compared with baseline (Figure 2c). The mean BCVA at baseline and months 1, 3, 6, and 12 were 0.76 ± 0.27, 0.66 ± 0.36, 0.61 ± 0.33, 0.62 ± 0.36, and 0.73 ± 0.46, respectively (Figure 2d). The mean BCVA tended to slightly improve until 6 months, but there were no statistical differences during the observation period (Figure 2d) (*p*-value < 0.05, Steel–Dwass test).

### 3.4. Additional Treatments

The additional anti-VEGF therapy until 12 months after IVR/tPA is shown in Table 2. Only 1 eye did not require any additional treatment and the other 24 eyes required additional anti-VEGF injections. In total, 14 eyes received IVR monotherapy, 5 eyes received IVA monotherapy, and 5 eyes switched from IVR to IVA. The mean total number of anti-VEGF injections was 3.6. Other treatments were additional IVR/tPA due to recurrence of SHRM (1 eye), vitrectomy due to vitreous hemorrhage (1 eye), and tPA and gas injection due to submacular hemorrhage (1 eye).

### 3.5. Change in SHRM and BCVAtatus

**Figure 3:**
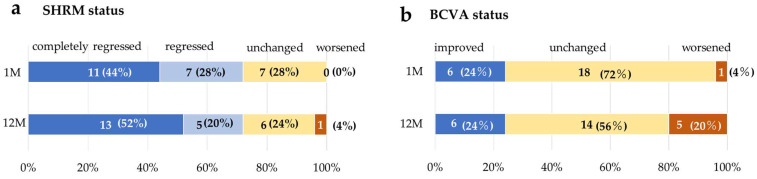
Changes in SHRM and BCVAtatus. The SHRM regressed in 18 eyes (72%) of the patients at 1 and 12 months; at month 12, SHRM worsened in 1 eye (**a**). The BCVA improved in 6 eyes (24%) and worsened in 1 eye (4%) at 1 month, improved in 6 eyes (24%) and worsened in 5 eyes (20%) at 12 months (**b**). M = month.

At month 1, SHRM completely regressed in 11 eyes (44%), reduced in 7 eyes (28%), and was unchanged in 7 eyes (28%). At month 12, SHRM completely regressed in 13 eyes (52%) [9 of 13 eyes (36%) had no recurrence after complete regression of SHRM at month 1; 4 eyes (16%) completely regressed by month 12], reduced in 5 eyes (20%) [2 eyes (8%) regressed once but recurred during follow-up], was unchanged in 6 eyes (24%), and worsened in 1 eye (4%) (Figure 3a).

The BCVA improved in 6 eyes (24%), was unchanged in 18 eyes (72%), and worsened in 1 eye (4%) at 1 month, and improved in 6 eyes (24%), was unchanged in 14 eyes (56%), and worsened in 5 eyes (20%) at 12 months (Figure 3b).

### 3.6. Distribution of BCVA at Month 12

**Figure 4:**
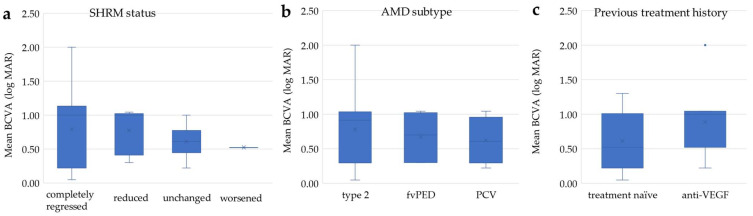
Distribution of BCVA at month 12. The BCVA at month 12 assessed by SHRM status (**a**), AMD subtype (**b**) and treatment history (**c**). There were no significant differences in BCVA among eyes with each groups SHRM status (Steel–Dwass test, *p* < 0.05), AMD subtype (Bonferroni’s test, *p* < 0.05) and previous treatment history (unpaired *t*-test, *p* < 0.05). – median, × mean, * outlier.

Figure 4a shows the BCVA assessed by SHRM status at 12 months. The mean BCVA at month 12 was 0.79 ± 0.59 in the completely regressed group, 0.77 ± 0.34 in the reduced group, 0.61 ± 0.26 in the unchanged group, and 0.52 in the worsened eye. The mean BCVA at month 12 was 0.78 ± 0.52 with type 2 MNV, 0.67 ± 0.36 with type 1 MNV with fibrovascular PED, and 0.62 ± 0.34 with PCV (Figure 4b) and 0.61 ± 0.44 with previous treatment group and 0.89 ± 0.46 without previous treatment (Figure 4c).

There were no significant differences in the BCVAs among eyes with each SHRM status (Steel–Dwass test, *p* < 0.05), AMD subtype (Bonferroni’s test, *p*-value < 0.05) and previous treatment history (unpaired *t*-test, *p*-value < 0.05).

### 3.7. Representative Case

**Figure 5:**
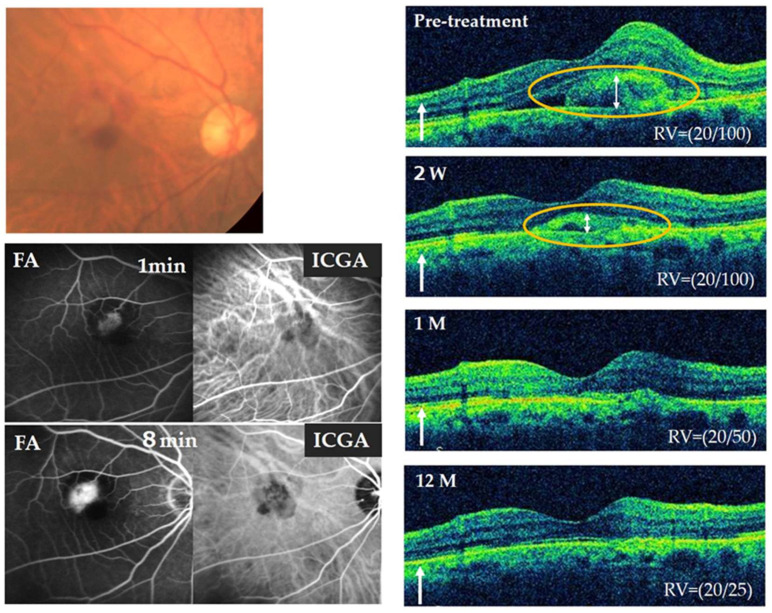
Neovascular age-related macular degeneration with macular neovascularization type 2 in an 85-year-old treatment-naïve male patient. The color fundus photograph in the right eye observed a well-demarcated gray lesion surrounded by a ring of marginal mild subretinal hemorrhage (**top left**). The FA in the early phase showed a well-demarcated hyperfluorescent lesion surrounded by a ring of slight hypofluorescence (fluorescence blocking) and in the late phase showed fluorescence leakage, while the ICGA depicted type 2 MNV surrounded with hypofluorescence spots derived fibrinous lesions and subretinal hemorrhage (**bottom left**). The OCT showed SHRM emanating through a break in the retinal pigment epithelium (RPE) and lying above the RPE suggesting a type 2 neovascular complex with marginal hemorrhage (orange circle) (**top right**). IVR/tPA combined therapy was performed as the initial treatment. SHRM was decreased 2 weeks after IVR/tPA (orange circle and white arrow). After 1 month, SHRM was regressed completely and maintained at month 12 without additional treatment (**right top to bottom**). BCVA improved from 20/100 to 20/50 at month 1 and 20/25 at month 12. W = week, M = month, RV = best corrected visual acuity in the right eye.

Figure 5 shows a representative case. An 85-year-old man was diagnosed with type 2 MNV in his right eye by fluorescein angiography (FA), Indocyanine green angiography (ICGA) and OCT. IVR/tPA combined therapy was performed as the initial treatment. A remarkable reduction in SHRM was observed 2 weeks after IVR/tPA. After 1 month, complete regression of SHRM was observed and the BCVA improved from 20/100 to 20/50. No recurrence of SHRM was observed till month 12 and the BCVA further improved up to 20/25 without additional treatment.

## 4. Discussion

In this study, we evaluate the impacts of the combination therapy with IVR/tPA in eyes with nvAMD accompanying SHRM. The IVR/tPA combination therapy dissolved the fibrin immediately. The SHRM decreased from an average thickness of 369 µm at baseline to 166 µm at 1 month; this effect was maintained at 165 µm at 12 months. At 12 months, the SHRM regressed completely in 52% of eyes and decreased in 20% of eyes. The mean BCVA in logMAR unit was 0.76 at the baseline and maintained at 0.73 at 12 months, and at 12 months, BCVA improved in 24% of eyes, maintained in 56% of eyes, and worsened in 20% of eyes.

SHRM is considered a poor prognostic factor for visual outcomes in patients with nvAMD [19,20,21,22]. The fact that SHRM was reduced in 72% of patients and vision was maintained in 80% of patients suggests that IVR/tPA may be effective in maintaining visual acuity in nvAMD patients. tPA is often used as an adjuvant to displace subretinal hemorrhages (especially submacular hemorrhage) caused by nvAMD or retinal macroaneurysm [24,25,26,27,28,29] in the ophthalmological field. Although displacement itself is mainly due to the tamponade effect of the gas, tPA is thought to play an ancillary role by dissolving hematoma or fibrin and causing a larger displacement of the hematoma. In our previous survey [34], many facilities in Japan used tPA in combination with gas rather than gas alone. Furthermore, our previous study has demonstrated the efficacy of IVR/tPA combination therapy was superior to the IVR monotherapy group at month 6 [30] in eyes with type 2 MNV.

We have focused on the role of tPA in angiogenesis and have examined its effects on angiogenesis in vivo and in vitro studies. Arai et al. [35] reported that while tPA has no direct impact on the vascular endothelial cells in vitro, tPA significantly reduced corneal neovascularization in a rabbit model. These results suggest that among the essential steps in angiogenesis, fibrin may play a pivotal role as a scaffold in the loose intercellular spaces around highly permeable vessels and that tPA may have an inhibitory effect on angiogenesis through its fibrinolytic and constrictive effects on the developing fibrovascular tissue.

Ozone et al. [36] reported that in an experimental laser-induced choroidal neovascularization (CNV) model in mice, intravitreal injection of tPA suppressed fibrin/fibrinogen expression, CNV leakage, and CNV volume without retinal toxicity. These results also suggested that fibrin may have a pivotal role as an alternative extracellular matrix that bridges the loosened intercellular space around highly permeable vessels and provides a scaffold for endothelial cells to migrate and proliferate and fibrinolysis by exogenous tPA may disrupt the fibrin scaffold and possibly interfere with migration and proliferation and the subsequent steps in angiogenesis. Therefore, tPA may have a potential role as an adjuvant therapy for CNV secondary to AMD.

**Figure 6:**
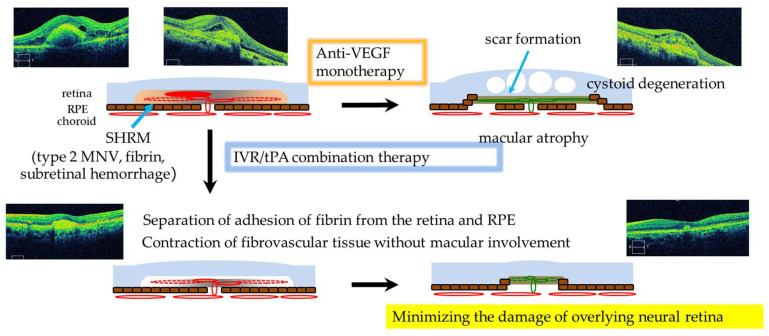
Possible roles of IVR/tPA in eyes with wet AMD accompanying SHRM. In nvAMD accompanying SHRM, anti-VEGF monotherapy suppresses the activity of CNV, fibrovascular scarring involves the outer retina and causes severe vision loss. The combination therapy with IVR/tPA separates the adhesion of fibrin from the retina and RPE and contracts fibrovascular tissue without involvement of the outer retina. Thus tPA minimizes the damage of overlying neurosensory retina. (Modified and quoted from [30]).

We hypothesize the following possible roles for IVR/tPA in eyes with nvAMD accompanying SHRM (Figure 6). SHRMs, including neovascular vessels, fibrin, and subretinal hemorrhage, are often adherent to the retina and RPE via permeable vessel-derived fibrin. Anti-VEGF monotherapy suppresses the activity of CNV. However, fibrovascular scarring involves the outer retina and causes irreversible damage with cystoid macular degeneration and severe vision loss. In contrast, the combination therapy with IVR/tPA may cause the fibrin to separate from the retina and RPE by dissolving the fibrin immediately after tPA administration and possibly result in contraction of the fibrovascular tissue without involvement of the outer retina. Consequently, tPA may minimize the damage of the overlying neural retina and decrease the risk of severe vision loss in nvAMD accompanying SHRM. Therefore, IVR/tPA should be performed before SHRM damages the neurosensory retina in the eyes both with and without previous treatment.

We used ranibizumab as an anti-VEGF agent combined with tPA. Klettner A et al. [37] reported that ranibizumab is not cleaved or functionally compromised by rtPA or plasmin, while aflibercept is cleaved and its VEGF-binding ability is reduced when coapplied with plasmin. Therefore, in this study, IVA was not used for the combination therapy. On the other hand, brolucizumab [7] and faricimab [8] have been newly approved for the treatment of nvAMD. The combination of these new options as well as aflibercept may be considered with the potential of further efficacy in the future.

Our study had several limitations that warrant consideration.

First, certain cases of PCV with both SMH and hard exudates were excluded because of the potential risk of exacerbating exudative changes with tPA fibrin dissolution. In addition, cases involving large SMHs affecting the fovea also were excluded, because pneumatic displacement with intravitreal injection of expandable gas and tPA is more appropriate. Moreover, patients with less potential for VA improvement or those at risk of worsening systemic diseases were excluded, because this treatment is unapproved status. The limited indications are important to make this therapy effective, but further studies are needed to determine the indications.

The second issue concerns the optimal concentration of tPA. We administered 40 kIU of tPA via vitreous injection, a dosage consistent with previous Japanese studies addressing macular edema associated with central retinal vein occlusion [32,33] or branch retinal vein occlusion [31] without significant reported complications. Another contributing factor to our decision was our institution’s longstanding practice of utilizing 40 kIU of tPA during intravitreal injection for the displacement of SMHs, which has not resulted in any discernible complications attributable to tPA. Nevertheless, further research is needed to determine the optimal tPA concentration.

In addition, the small sample size, short follow-up period, and retrospective nonrandomized trial design limit the robustness of our findings. Future studies with larger sample sizes and more extensive analyses are warranted. Further, our study did not compare IVR to IVR monotherapy. Larger randomized controlled trials should assess the long-term visual outcomes of combination therapy comprehensively.

## 5. Conclusions

The combination therapy with IVR/tPA for nvAMD with SHRM may help preserve vision by regression of SHRM.

## Figures and Tables

**Table 1:** Patient characteristics.

Cases	25 Eyes of 25 Patients	
Gender	16 men and 9 women	
Mean age	76.7 ± 9.8 years old	
Subtype	Type 2 MNV	16 eyes
	Type 1 MNV with fibrovascular PED	5 eyes
	PCV	4 eyes
Previous treatment history	Treatment-naïve	15 eyes
	Anti-VEGF therapy and/or PDT	10 eyes

**Table 2:** Additional anti-VEGF therapy.

Cases	25 Eyes of 25 Patients	
None additional treatment		1 eye
Type of anti-VEGF	IVR mono therapy	14 eyes
	IVA mono therapy	5 eyes
	Switch from IVR to IVA	5 eyes
Mean number of injections		3.6 ± 2.2 times

## Data Availability

Researchers can contact Aki Kato, MD, PhD, (akikato@med.nagoya-cu.ac.jp) for details of the protocol and results.
